# Clinical efficacy of Neoadjuvant Chemotherapy combined with Laparoscopic Surgery in patients with Middle and Low Rectal Cancer and its Effect on serum VEGF Levels and quality of life

**DOI:** 10.12669/pjms.39.6.6763

**Published:** 2023

**Authors:** Wei Hou, Xin Song, Qingfang Pan, Sukun Liu, Qinglian Zhao, Dan Hong

**Affiliations:** 1 Wei Hou, Department of Radiotherapy, Baoding NO.1 Central Hospital, Baoding 071000, Hebei, China; 2 Xin Song Endoscopy Room, Baoding Dawu Hospital, Baoding 072550, Hebei, China; 3 Qingfang Pan Department of Medical Oncology, Chinese People’s Liberation Army No.82 Group Hospital, Baoding 071000, Hebei, China; 4 Sukun Liu Department of Medical Oncology, Affiliated Hospital of Hebei University, Hebei Key Laboratory of Cancer Radiotherapy and Chemotherap, Baoding 071000, Hebei, China, Department of Radiotherapy, Baoding NO.1 Central Hospital, Baoding 071000, Hebei, China; 5 Qinglian Zhao, Department of Radiotherapy, Baoding NO.1 Central Hospital, Baoding 071000, Hebei, China; 6 Dan Hong, Department of Radiotherapy, Baoding NO.1 Central Hospital, Baoding 071000, Hebei, China

**Keywords:** Neoadjuvant Chemotherapy, Laparoscopic Surgery, Rectal Cancer, VEGF level, Quality of life

## Abstract

**Objective:**

To observe the clinical efficacy of neoadjuvant chemotherapy combined with laparoscopic surgery in patients with middle and low rectal cancer and its effect on serum VEGF level and quality of life.

**Methods:**

Retrospective analysis was performed on 80 patients with middle and low rectal cancer admitted to Baoding No.1 Central Hospital from June 2018 to June 2020.They were randomly divided into two groups. Patients in the control group underwent laparoscopic radical rectal cancer surgery, while those in the experimental group underwent neoadjuvant chemotherapy before surgery. The differences of various surgical indicators between the two groups were compared. The incidence of surgical complications, the serum VEGF levels and the improvement of quality of life were compared. The differences in local recurrence, metastasis and overall survival with in two years after surgery were compared.

**Results:**

The various surgical indicators of the experimental group were significantly better than the control group (p<0.05). After treatment, the VEGF levels in the experimental group were significantly lower than those in the control group (p=0.00), while the SF-36 score was significantly higher than that of the control group (p=0.00). The total incidence of surgical complications in experimental group was significantly lower (p=0.03), the local recurrence rate was significantly lower (p=0.02), and the overall survival rate was significantly higher than that in control group (p=0.04).

**Conclusion:**

Neoadjuvant chemotherapy combined with laparoscopic surgery is superior to direct surgery alone in the treatment of middle and low rectal cancer and it needs to be promoted.

## INTRODUCTION

Rectal cancer is one of the more common malignant tumors of the digestive tract.^1^ Tumors in some patients with advanced stages may invade the surrounding organs such as the bladder and urethra.^2^ Surgical removal of the tumor is currently the main preferred method for the treatment of rectal cancer in clinical practice.^3^ However, such a surgical method has a low success rate of anal preservation, or is associated with anal dysfunction after anal preservation that affects patients’ quality of life due to the low location of rectal cancer, especially the proximity of the middle and low tumors to the anal sphincter. Neoadjuvant chemoradiotherapy is an adjuvant chemoradiotherapy regimen given to patients with malignant tumors before surgery, promoting the function of reducing preoperative tumor load and improving overall therapeutic effect.^4^ It was reported in a study by Pinto et al^5^ That patients receiving neoadjuvant radiotherapy may have increased serious defecation times, reduced fecal control ability and reduced quality of life due to radiation enteritis, and some patients may even need permanent fistula due to anal incontinence or stricture.^6^ In this study, neoadjuvant chemotherapy combined with laparoscopic surgery was utilized to treat patients with middle and low rectal cancer, now the specific report is as follows.

## METHODS

Retrospective analysis was performed on 80 patients with middle and low rectal cancer admitted to Baoding NO.1 Central Hospital from June 2018 to June 2020. The patients were randomly divided into two groups: the control group and the experimental group, with 40 cases in each group. No significant difference of general data between the two groups, which was comparable (Table-I).

**Table-I:** Comparative analysis of general data between the experimental group and the control group (*χ̅*±*S*) n=40.

Indicators	Experimental group	Control group	t/χ^2^	P
Age (years old)	55.83±6.04	53.89±6.18	1.42	0.16
Male (%)	27 (%)	25 (%)	2.22	0.64
Pathological type				
Adenocarcinoma (%)	22 (%)	23 (%)	0.05	0.82
Squamous cell carcinoma (%)	10 (%)	9 (%)	0.07	0.79
Undifferentiated cancer (%)	6 (%)	7 (%)	0.09	0.76
Others (%)	2 (%)	1 (%)	0.35	0.57
Distance from the tumor to the anus (cm)	7.03±1.14	6.75±2.08	0.75	0.46
Tumor staging				
T2 (%)	11 (%)	12 (%)	0.06	0.81
T3 (%)	22 (%)	20 (%)	0.20	0.65
T4a (%)	7 (%)	8 (%)	0.08	0.76

P<0.05.

### Ethical approval

The study was approved by the Institutional Ethics Committee of Baoding NO.1 Central Hospital dated (No.: 2021015; date: June 14 2022), and written informed consent was obtained from all participants.

### Inclusion criteria

- Patients with preoperative CT, MRI and other imaging examinations and pathological examinations that meet the diagnostic criteria for rectal cancer and T stage ≤ T4a;^7^
- Patients who have not received regular treatment before the initial diagnosis;
- Patients less than 70 years old; - Patients whose tumor location is less than 10 cm from the anal margin;
- Patients whose expected survival time is ≥ six months;
- Patients with no distant metastasis and no obvious surgical contraindications from preoperative examination; - Patients without contraindications to the drugs used in this study.

### Exclusion criteria

- Patients with malignant tumors in other parts;
- Patients with severe cardiopulmonary dysfunction who cannot tolerate surgery;
- Patients with severe complications and unable to tolerate surgery.

### Treatment methods

Patients in the control group underwent laparoscopic radical resection of rectal cancer within one month after diagnosis, and continued chemotherapy or chemoradiotherapy according to stage after surgery. Surgical methods:^8^ Patients were subjected to general anesthesia, the lithotomy position was taken. Artificial pneumoperitoneum was established and put into laparoscope. Incisions were made on the flat umbilicus of the left mid-clavicular line and the outer edge of the right lower rectus abdominis, respectively, and trocar and endoscopic instruments were inserted respectively. Patients with tumors more than 6-cm from the anal margin underwent transabdominal anterior rectal cancer resection.

The bare intestinal wall was free, and the closed bowel about two cm at the lower edge of the tumor was cut off. An incision about five cm in length was made in the middle of the patient’s lower abdomen, the tumor was removed with the help of a protective sleeve, and the bowel was removed 10 cm near the mouth of the tumor. A stapler was built into the proximal intestinal cavity, the other stapler was placed into the anus and aligned and anastomosed with the intra-abdominal stapler. If the tumor is less than or equal to six cm from the anal margin, transabdominal perineal resection should be performed. Extraperitoneal tunnel colostomy was performed on the left lower abdomen, and the perineal surgery method was the same as traditional surgery.

Patients in the study group underwent neoadjuvant chemotherapy before surgery. The specific regimen was as follows: capecitabine (1000 mg/m^2^) was orally administered for two weeks, followed by withdrawal for one week; Oxaliplatin injection 100 mg/m^2^ was injected intravenously on the 2nd day. Surgery was performed eight weeks after the completion of neoadjuvant radiochemotherapy.

### Observation indicators:

### Surgical indicators

The differences of operation time, intraoperative blood loss, postoperative hospitalization time, gastrointestinal function recovery time, surgical resection success rate and anus preservation rate were observed between the two groups.

### Surgical complications

The incidence of intestinal obstruction, incision infection, anastomotic leakage. Serum VEGF levels: the venous blood of patients were collected in all cases under fasting condition in the morning before treatment and six and 12 months after surgery respectively to detect serum VEGF levels.

The Quality of Life table (SF-36)9 was used to assess the quality of life of the two groups before treatment and at six and 12 months after treatment, with a score range of zero to 100 points. The higher the score, the better the quality of life. All patients were followed up for two years, and the differences in the local recurrence, metastasis and overall survival rate of the two groups within two years after surgery were evaluated.

### Statistical analysis

All the data were statistically analyzed by SPSS 20.0 software, and the measurement data were expressed as (*χ̅*±*S*). Two independent sample t-test was used for inter-group data analysis, paired t test was used for intra-group data analysis. The rate comparison was performed using c^2^ test. P<0.05 indicates a statistically significant difference.

## RESULTS

The experimental group was significantly better than the control group in terms of operation time, intraoperative blood loss, tumor resection success rate, anus preservation rate, postoperative hospitalization time, and anal exhaust time (p<0.05) (Table-II). The total incidence of surgical complications in the experimental group was 12.5%, which was significantly lower than the 32.5% in the control group (p=0.03) (Table-III).

**Table-II:** Comparison and analysis of surgical conditions between the two groups (*χ̅*±*S*) n=30.

Group	Operation time (min)*	Blood loss (ml)*	Hospitalization time (d)*	Anal exhaust time (d)*	Tumor resection success rate (%)*	Anus preservation rate (%)*
Experimental group	103.76±13.17	77.36±24.13	16.82±2.36	4.37±1.32	40 (100%)	37
Control group	124.34±15.26	113.35±25.82	23.34±3.47	6.79±1.49	36 ()	30
*t*/*c*^2^	2.38	6.44	9.83	7.68	4.21	4.50
*P*	0.02	0.00	0.00	0.00	0.04	0.03

* p<0.05.

**Table-III:** Comparative analysis of surgical complications between the two groups (*χ̅*±*S*) n=40.

Group	Incision infection	Intestinal obstruction	Anastomotic leakage	Total incidence
Experimental group	2	2	1	5 (12.5%)
Control group	3	4	6	13 (32.5%)
*c* ^2^				4.59
*P*				0.03

p<0.05.

The VEGF levels in the experimental group were significantly lower than those in the control group at six and twelve months after treatment (p=0.00) (Table-IV). The SF-36 scores of the two groups increased at six months and twelve months after treatment (p=0.00). The SF-36 score of the experimental group at six months and twelve months after treatment was significantly higher than that of the control group (p=0.00) (Table-V).

**Table-IV:** Comparative analysis of serum VEGF (ng/l) levels before and after treatment between the two groups (*χ̅*±*S*) n=40.

Group	Before treatment	6 months after treatment*	12 months after treatment*	F	P
Experimental group	422.58±43.24	326.27±37.45	280.75±25.47	17.87	0.00
Control group	431.74±43.82	386.71±36.39	336.08±31.48	11.31	0.00
t	0.94	7.32	8.64		
P	0.35	0.00	0.00		

* p <0.05.

**Table-V:** Comparative analysis of the improvement of the quality of life between the two groups before and after treatment (*χ̅*±*S*) n=40.

Group	Before treatment	6 months after treatment*	12 months after treatment*	F	P
Experimental group	40.35±3.76	66.32±3.46	80.53±4.76	37.85	0.00
Control group	41.74±3.27	53.46±3.39	76.06±3.48	32.46	0.00
t	1.76	16.79	4.79		
P	0.08	0.00	0.00		

* p <0.05.

The local recurrence rate of the experimental group was 2.5%, significantly lower than the 17.5% of the control group (p=0.02). The overall survival rate was 85% in the experimental group, significantly higher than the 65% in the control group (p=0.04) (Table-VI).

**Table-VI:** Comparative analysis of the follow-up results between the two groups (*χ̅*±*S*) n=40.

Group	Local recurrence (%)*	Transfer (%)	Overall survival rate (%)*
Experimental group	1 (2.5%)	3 (7.5%)	34 (85%)
Control group	7 (17.5%)	5 (12.5%)	26 (65%)
c^2^	5.00	0.55	4.27
P	0.02	0.46	0.04

* p<0.05.

## DISCUSSION

Our study showed that the SF-36 score of the experimental group at six months and twelve months after treatment was significantly higher than that of the control group. The local recurrence rate of the experimental group was significantly lower than that of the control group, and the overall survival rate was significantly higher. It was also shown in our study that patients receiving combined neoadjuvant chemotherapy were significantly better than those in the control group in terms of operation time, intraoperative blood loss, tumor resection success rate, anus preservation rate, postoperative hospital stay, anal exhaust time and other indicators.

Rectal cancer is a malignant tumor of the digestive tract with a very high incidence worldwide.^10^ In the past, surgical resection of the tumor was the preferred clinical treatment for patients with locally advanced rectal cancer. However, such a surgical scheme is often associated with a high local recurrence rate.^11^ Before a surgery is being performed, the blood supply of rectal tumors was sound, and the tumor cells were highly sensitive to hypoxia and chemoradiotherapy. Neoadjuvant chemoradiotherapy promotes various effects, such as reducing tumor, degrading and downgrading it, increasing complete resection rate, possibly increasing anus preservation rate^12^, and reducing the difficulty of surgery and increasing the success rate of surgery. The total complication rate of the experimental group was significantly lower than that of the control group. VEGF can induce endothelial cell proliferation, and its high expression is related to tumor growth, metastasis and infiltration.^13^ According to Wu et al.^14^, the level of serum VEGF in patients with metastatic colorectal cancer decreased significantly after chemotherapy, indicating that the serum VEGF level of the patients is related to the efficacy of chemotherapy. It was believed by Nussbaum et al.^15^ that the detection of serum VEGF levels of patients with rectal cancer may have extremely important clinical significance in guiding the treatment and prognosis of patients with metastatic colorectal cancer. It was suggested in our study that the VEGF level of the experimental group after treatment was significantly lower than that in the control group. With the increase of the number of rectal cancer survivors, more and more patients taken sphincter-sparing surgery have to live with a potentially impaired quality of life.^16^ Neoadjuvant chemotherapy can reduce the clinical staging of tumors, reduce the scope of resection, and be more beneficial to postoperative control.^17^

A study involving 430 patients indicated that compared with conventional surgical treatment, the rate of stoma dysfunction in the neoadjuvant chemotherapy group was significantly lower than that in the conventional treatment group.^18^ Matsutani et al.^19^ believed that neoadjuvant therapy for locally advanced rectal cancer, especially radiotherapy, can induce the activation of local immune status, reduce the local recurrence rate and the chance of distant metastasis^20^, as well as alleviate the pain of postoperative patients and improve QOL.

### Limitations

It includes a small number of sample cases were included in the study with a short follow-up time. In addition, only neoadjuvant chemotherapy was included in the study for comparison with conventional laparoscopic surgery, while no systematic comparative study has been conducted on the role of neoadjuvant radiotherapy and neoadjuvant chemotherapy in surgery.

## CONCLUSION

To put it in a nutshell, neoadjuvant chemotherapy combined with laparoscopic surgery offers a variety of advantages in the treatment of patients with middle and low rectal cancer, such as short operation time, less blood loss, high success rate, rapid recovery, low recurrence rate, significantly reduced VEGF levels, and better prognosis than those who underwent surgery directly.

### Authors’ Contributions:

**WH and XS:** Designed this study, prepared this manuscript, are responsible and accountable for the accuracy and integrity of the work.

**QP and SL:** Collected and analyzed clinical data.

**QZ and** **DH:** Participated in acquisition, analysis, or interpretation of data and draft the manuscript.
